# Neural, physiological, and psychological markers of appetitive conditioning in anorexia nervosa: a study protocol

**DOI:** 10.1186/s40337-022-00546-5

**Published:** 2022-05-10

**Authors:** Stuart B. Murray, Tomislav D. Zbozinek, Michelle Craske, Reza Tadayonnejad, Michael Strober, Ausaf A. Bari, John P. O’Doherty, Jamie D. Feusner

**Affiliations:** 1 Department of Psychiatry and Behavioral Sciences, University of Southern California, 2250 Alcazar Street, Los Angeles, CA 90033 USA; 2 Division of Humanities and Social Sciences, California Institute of Technology, Pasadena, CA USA; 3 Department of Psychology, University of California, Los Angeles, Los Angeles, CA USA; 4 Department of Psychiatry and Biobehavioral Sciences, University of California, Los Angeles, Los Angeles, CA USA; 5 Computation and Neural Systems Program, California Institute of Technology, Pasadena, CA USA; 6 Department of Neurosurgery, University of California, Los Angeles, Los Angeles, CA USA; 7 Centre for Addiction and Mental Health, Toronto, Canada; 8 Department of Psychiatry, University of Toronto, Toronto, Canada

**Keywords:** Anorexia nervosa, Eating disorders, Reward, Appetitive conditioning, fMRI

## Abstract

**Background:**

Anorexia nervosa (AN) is a chronic and disabling psychiatric condition characterized by low hedonic drive towards food, and is thought to be inclusive of altered dimensions of reward processing. Whether there exists a fundamental aberrancy in the capacity to acquire and maintain de novo hedonic associations—a critical component of hedonic responding—has never been studied in AN.

**Methods:**

This multi-modal study will employ a 2-day Pavlovian appetitive conditioning paradigm to interrogate the (1) acquisition, (2) extinction, (3) spontaneous recovery and (4) reinstatement of appetitive learning in adolescents and young adults with AN. Participants will be 30 currently ill, underweight individuals with AN; 30 weight-restored individuals with AN; and 30 age-matched healthy controls, all aged 12–22 years. All subjects will undergo clinical assessment, followed by the 2-day appetitive conditioning task during which fMRI, pupillometry, heart rate deceleration, and subjective ratings will be acquired.

**Discussion:**

This study will be the first to interrogate appetitive conditioning in AN—a disorder characterized by altered hedonic responding to food. Results will help establish objective biomarkers of appetitive conditioning in AN and lay the groundwork for developing novel lines of treatment for AN and other psychiatric disorders involving diminished ability to experience pleasure and reward.

*Trial registration*: Pending.

**Intended registry:**

Clinicaltrials.gov.

**Supplementary Information:**

The online version contains supplementary material available at 10.1186/s40337-022-00546-5.

## Background

Anorexia nervosa (AN) is a disabling, often chronic, and potentially life-threatening eating disorder which is characterized by self-imposed starvation, emaciation, intense fear of weight gain, and a marked disturbance in how one’s body shape and weight is experienced [1]. AN demonstrates the greatest mortality rate of any psychiatric illness, with a crude mortality rate of 5.6% [2]. Even in nonlethal presentations, multi-systemic organ damage, bone disease, and structural and functional brain impairment are commonplace [3], which cumulatively render functional impairments comparable to those seen in schizophrenia [4] and autism [5]. Despite decades of investigation, the benefits of specialized treatments remain limited [6]. Remission rates typically range from 0 to 25% for adult presentations and approximately 15–33% for adolescent presentations [7], and more than half of those afflicted with AN still meet diagnostic criteria more than 2 decades after illness onset [8]. Importantly, the pathophysiology of AN is incompletely understood, stymying efforts towards improved, precision treatments. In short, treatment research has stalled, and the need to explicate the pathogenesis of AN cannot be overstated.

Owing to the centrality of food restriction, despite its near universal hedonic properties [9], much research investigating potential pathogenic mechanisms in AN has explored dimensions of reward-related processes. Cumulatively, research has pointed to aberrant experiences of reward; this includes the phenomenological observations, and self-reports, of diminished hedonic responses to typically hedonic cues [10, 11]. This is perhaps most evident in the context of food cues. Those with AN (1) rate olfactory, visual, and taste cues as markedly *less* pleasant than do healthy controls [12, 13]; (2) report increased *negative* affect after meals [14]; and (3) report food consumption to be *aversive*, rather than hedonic [15, 16]. Importantly, diminished hedonia such as in social situations is evident beyond the confines of food consumption among those with AN, with evidence suggesting a reduced drive to pursue novelty and fun [17, 18] and unease in social and sexual relationships [19, 20], which persists even after remission. This accords with the developmental history of those with AN, which is foreshadowed by reserve, caution, regimentation, and a lowered drive for pleasure and novelty seeking [10, 11, 21]. Thus, reduced responding to typically appetitive rewards, which, prima facie, could be the result of an atypical capacity to abstain from these rewards, may alternatively indicate a diminished capacity for hedonic responses. For either alternative, disturbance in appetitive responding is a potentially salient contributor to overall morbidity in terms of not only restricted food consumption, but impaired social interactions and overall diminished quality of life.

Alterations in hedonic responding are also reflected in psychophysiological markers. For instance, upon exposure to palatable food cues, those with AN demonstrate reduced zygomatic and greater corrugator facial muscle activity [22, 23], suggesting less positive and more negative affective states. Similarly, in social contexts, those with AN typically demonstrate avoidance of positive social cues [24, 25] and less positive facial affect and more negative facial affect to positive social cues [26, 27]. In concert, studies assessing dopaminergic function—a critical neurotransmitter relating to reward learning and hedonic responding—suggest trait level abnormalities in those with AN. For instance, reduced concentrations of CSF homovanillic acid—a dopamine metabolite—have been found in those with AN, even after recovery [28], and greater dopamine receptor availability has been noted in the ventral striatum of those with AN [29].

Underlying structural abnormalities in the brain’s reward circuitry have also been observed in AN, which extends to gray matter structures and white matter fiber tracts. Gray matter abnormalities are characterized by reduced volume in key nodes of the brain’s reward circuit, including the ventral striatum and orbitofrontal cortex (OFC) [30, 31], and aberrant connectivity of white matter tracts between these regions [32, 33]. Moreover, preliminary evidence suggests that disturbed frontocortical-accumbal connectivity is associated with the severity of AN symptomatology [32].

Despite the consistency of these multimodal findings in suggesting disturbed hedonic responding in AN, findings from functional MRI studies are surprisingly varied. In the context of food cues, studies have generally noted reduced cortico-striatal activity among those with AN [34, 35], which extends to the taste, smell, and sight of palatable foods [34, 36–38]. In contrast, some studies of AN have illustrated *hyperactivity* in reward-related regions, such as the ventral striatum [39] and amygdala [40] in response to palatable-calorie food cues, despite those with AN implicitly and explicitly stating ‘liking’ and ‘wanting’ these foods less than controls [12]. At the same time, elevations in prefrontal regions including the medial prefrontal cortex and dorsolateral prefrontal cortex following exposure to palatable food cues has driven several hypotheses suggesting elevated top-down inhibition of reward processing [41–43]. In keeping with behavioral data, aberrant neural response to hedonic cues extends beyond food cues. For instance, those with AN show abnormal cortico-striatal activity in response to monetary rewards [44], which accords with behavioral data suggesting a reduced proclivity towards valuing and pursuing immediate monetary gain [45].

Paradoxically, those with AN describe the restriction of food consumption, and feeling hungry, as intensely rewarding [10, 11]. This raises the intriguing possibility that dimensions of reward processing may remain intact but have nearly opposite associations as those in healthy individuals. That is, cues and states typically described as rewarding among those with AN (i.e., food restriction, hunger) are those typically described as *aversive* among those without AN [10, 11]. Contributing to this notion, the portrayal of self-starvation and relentless exercise as positive experiences and desirable lifestyle choices are themes that emerge from interviews of those with AN [46, 47]. This is consistent with data demonstrating elevated ventral striatum activity following exposure to images of thin people [48, 49] and elevated OFC activity observed in response to low calorie foods, which is exacerbated by fasting levels of acylated ghrelin [50]. This suggests that hunger may amplify the reward processing of low-calorie foods.

What could account for this subjective experience of the hunger state and low body weight as rewarding, however, remains unknown. It is possible that what is subjectively ‘rewarding’ is the avoidance of the perceived threat of food intake or normative body mass. Nevertheless, the mechanisms to explain the divergence of reward system responses are likely not simple, necessitating an understanding at higher levels of complexity, and across multiple units of analysis [51].

Whether there exists a fundamental aberrancy in hedonic processes in AN is unknown and has been minimally studied. Existing studies have exclusively interrogated reward circuitry in the circumscribed contexts of decision making, cognitive processes and prediction error around cues with an assumed incentive salience (i.e., money, sucrose) [52–54]. Yet, significant gaps remain in our understanding of key reward-related constructs of hedonic processing and appetitive behavioral learning, and no studies to date have interrogated the capacity for de novo learning of hedonic associations in AN. Ascertaining the integrity of appetitive learning systems is of critical importance in AN, for which an ongoing indifference to palatable food consumption often persists despite specialized treatments [55, 56] and may contribute to its high rates of relapse [57]. Illness remission across *all* treatment modalities is predicated on a therapeutic ability to enhance the incentive salience of food cues and promote approach behaviors and greater food consumption as well as help remediate impairments in social functioning [18]. As such, an increased understanding of appetitive learning in AN, and its decay and reinstatement, will directly inform efforts to augment patient outcomes by altering the incentive salience of recovery-congruent cues and food-related approach behaviors.

Appetitive Pavlovian conditioning paradigms offer a well-validated methodology to interrogate how positively-valenced associations are acquired, extinguished, and reinstated [58]. Perturbations have been linked to disease-specific symptomatology; appetitive conditioning studies in depression—a disorder highly comorbid and genetically correlated with AN [59]—illustrate abnormal activity in the amygdala, ventral striatum (VS), and orbitofrontal cortex (OFC), which results in a failure to generate positive associations to typically hedonic cues [60]. Appetitive conditioning studies frequently employ palatable food cues (i.e., chocolate, sucrose) as the unconditioned stimulus (US). Importantly, however, the use of tastants in AN populations is problematic as it is conflated with the existing aversive associations to palatable food tastants. Disorder-neutral USs are thus warranted.

In addition, the potentially confounding effects of weight and nutritional status are important considerations. Animal studies suggest that food restriction and weight loss drive a sensitization of brain reward pathways [61–63] and dopaminergic neuronal activity [64]. This adaptive sensitization of D1 and D2/D3 receptors in response to food restriction is thought to stimulate motivation to approach food [65]. In keeping, the dopamine-mediated prediction error response is elevated in the NAcc, head of the caudate, and insula in underweight adolescents with AN [52], although this appears to normalize upon weight restoration [66]. In addition, some of the volumetric abnormalities in gray matter structures in the reward circuit are thought to normalize after weight restoration [67]. Current consensus in AN research advocates separating underweight from weight-restored patients with AN when unraveling the potential characteristics of the AN phenotype from state-specific effects of starvation [10].

With these considerations, we designed the present multimodal Pavlovian appetitive conditioning study, in underweight and weight-restored AN, to index the acquisition, extinction, spontaneous reinstatement, and recovery of associative reward learning—a key hypothesized mechanism in the pathogenesis and maintenance of AN. We will study these phenomena across multiple domains of analysis: subjective experience (self-report), physiology (heart rate deceleration and pupillary dilation), and neural response (brain activity in reward systems). Our conditioning paradigm employs positively-valenced, socially rewarding yet symptom-neutral (avoiding stimuli to which they may have been aversively conditioned) infant laughter sounds, which adolescents with AN typically rate positively (see Additional file 1). This study aims to assess in adolescent and transition age youth with restrictive type AN (AN-R) and healthy controls (1) the rate of association of a neutral cue with a paired hedonic outcome, (2) the rate of the decay of this association upon cessation of cue co-pairing, (3) the rate of spontaneous recovery (i.e., the increase in CS responding, after extinction, with the passage of time) and reinstatement (i.e., the increase in CS responding after extinction, following the presentation of the US alone) of this association upon cue exposure 24 h later, and (4) the patterns of physiological (heart rate deceleration, pupil dilation) and neural activation (fMRI) associated with acquisition, decay and reinstatement of this reward learning. In discerning whether any perturbations in reward learning represent state-specific effects of starvation or stable features of AN, this study will include underweight restricting-type AN (AN-R) patients, weight restored AN-R (WRAN-R) patients, and age-matched healthy controls. Further, with empirical findings noting stable sex differences in associative learning [68], the proposed study will include only female participants.

We hypothesize that all three groups will (1) demonstrate appetitive conditioning effects to the CS+ (the CS paired with the US) during the acquisition phase of the conditioning paradigm relative to the CS− (the CS *not* paired with the US), as evidenced by: positive experience and expectancy ratings, heart rate deceleration, pupillary dilation; and neural activation in the ventral striatum (node of the cortico-striatal circuit involved in appetitive conditioning) for the CS+ versus CS− contrast [58, 69]. Further, we predict that (2) across all groups, heart rate deceleration and pupillary dilation will moderate the relationship between ventral striatum activity and positive experience ratings. Further, we will ascertain whether (3) differences exist between AN groups (AN-R and WRAN-R) versus healthy controls in the rate of (a) acquisition, as well as (exploratory) (b) extinction, (c) spontaneous recovery, and (d) reinstatement of learned appetitive associations across neurological, physiological and self-report data. Moreover, and in delineating the effects of starvation among those with AN, we hypothesize that (4) those with AN-R, relative to those with WRAN-R, will demonstrate lower mean positive experience ratings, reduced heart rate deceleration, reduced pupillary dilation, and reduced neural activity for the CS+ − CS− contrast in the ventral striatum. We will also explore correlations between subjective experience ratings and both neural activity and physiological signals, within and across groups, to determine if AN (in the acute and/or weight restored state) is marked by a disconnect between subjective experience and neural and physiological phenomena.

## Methods

### Reproducibility

The trial protocol is openly available at (https://osf.io/nmgc2/). Moreover, this study will be prospectively registered with ClinicalTrials.gov prior to the collection of any data, and upon completion of data collection, summary data will be made publicly available. In addition, analytic scripts will be openly available at (https://osf.io/nmgc2/).

### Participants

Participants will be female adolescents and transitional age youth (aged 12–22 years) with a DSM-5 diagnosis of AN-R who are (1) currently underweight (N = 30), (2) weight-restored (WRANR) (N = 30), and (3) age-matched healthy controls (N = 30). In delineating underweight vs. weight restored AN-R, a weight inclusion criterion of < 87% of age-, height-, and sex-adjusted expected weight will be upheld for the underweight AN group [70]. Control participants scoring higher than two standard deviations from community norms on measures of ED pathology (i.e., > 2 on the Global Eating Disorders Examination score [71]) will be excluded. To obtain a clinically representative sample, we will not exclude comorbid anxiety and depressive disorders. All participants must have the ability to read and speak English fluently, and anyone demonstrating (1) medical instability, (2) a change in dose of psychotropic medication over the previous 4 weeks, (3) anyone taking antipsychotic medications, or (4) any contraindications for MRI, will be excluded. Recruitment will take place in the Greater Toronto and Hamilton, Ontario Area (> 7.35 million), with multiple University-based and public and private eating disorder treatment programs. Community recruitment will include social media, online advertisements, and flyers.

### fMRI appetitive conditioning paradigm

A 2-day differential Pavlovian conditioning paradigm widely used in human conditioning research will be used in this study. The unconditioned stimulus (US) will be infant laughter sounds, owing to the strong positive valence of infant laughter sounds [72], and our pilot data showing the positive self-reported valence of this sound among adolescents with AN (see Additional file 1). Prior to conditioning and owing to inter-individual variation in the degree to which each infant laughter cue is perceived as positive, participants will be exposed to a set of 18 baby laughter cues; the top 4 most positive rated cues for each individual will serve as the US in the conditioning paradigm. The conditioned stimuli (CSs) in this paradigm will be two distinctly colored geometric shapes. The US (the 4 most positively rated cues of infant laughter) will be delivered binaurally via noise canceling headphones (500 ms, 80dBA) and will co-terminate with the CS+. Notably, despite background MR scanner noise, infant laughter cues have been shown to elicit high magnitude BOLD signal changes in reward-related regions [73].

**Fig. 1:**
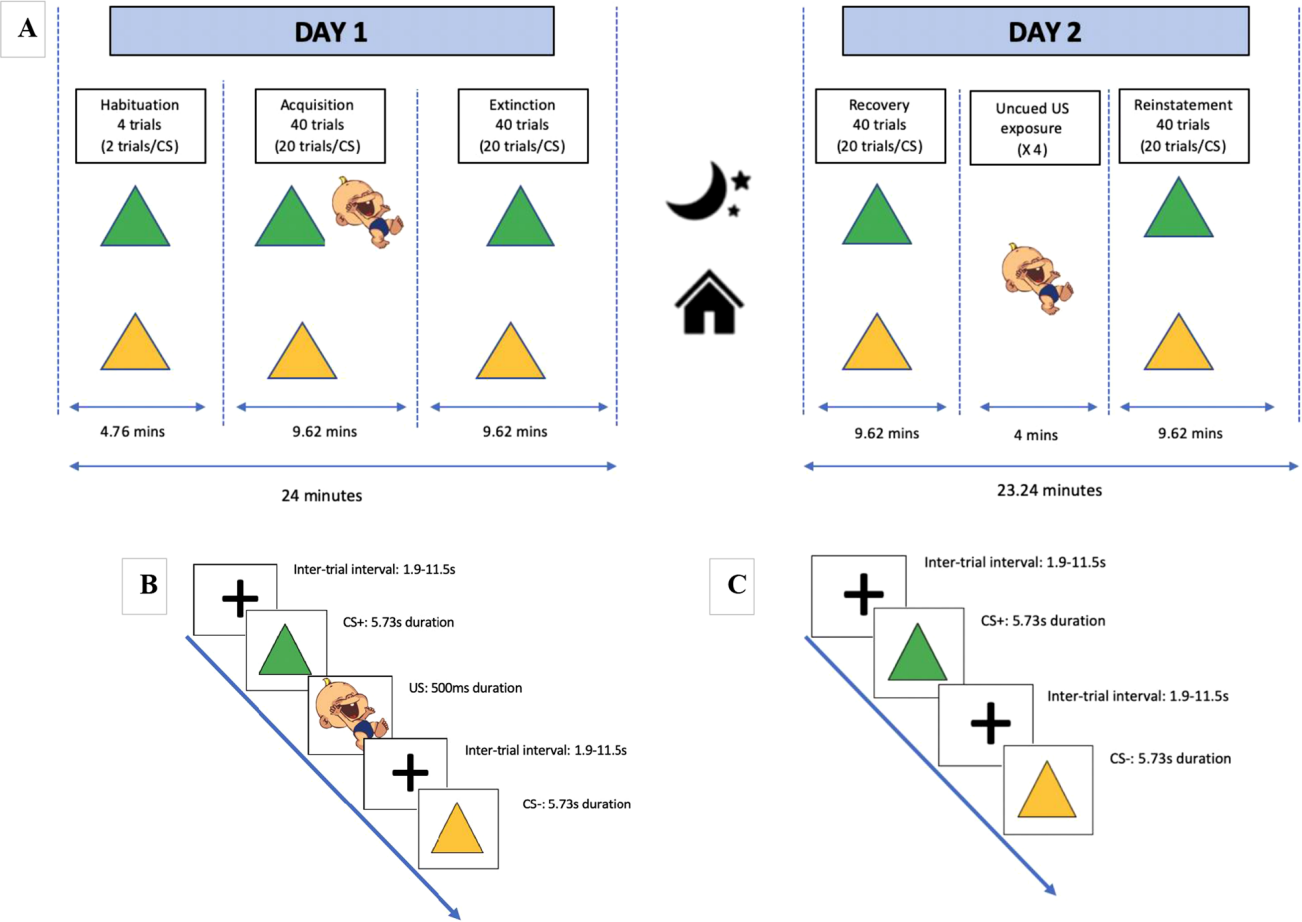
**A** An overview of the 2-day appetitive conditioning paradigm, the temporal sequencing of task phases, and the overall timing parameters of each phase. **B** represents an overview of cue sequencing for the acquisition phase, when the CS+ is paired with the US, and **C** represents an overview of cue sequencing for the habituation, extinction, recovery, and reinstatement phases, when the CS+ is presented without the US

**Fig. 2:**
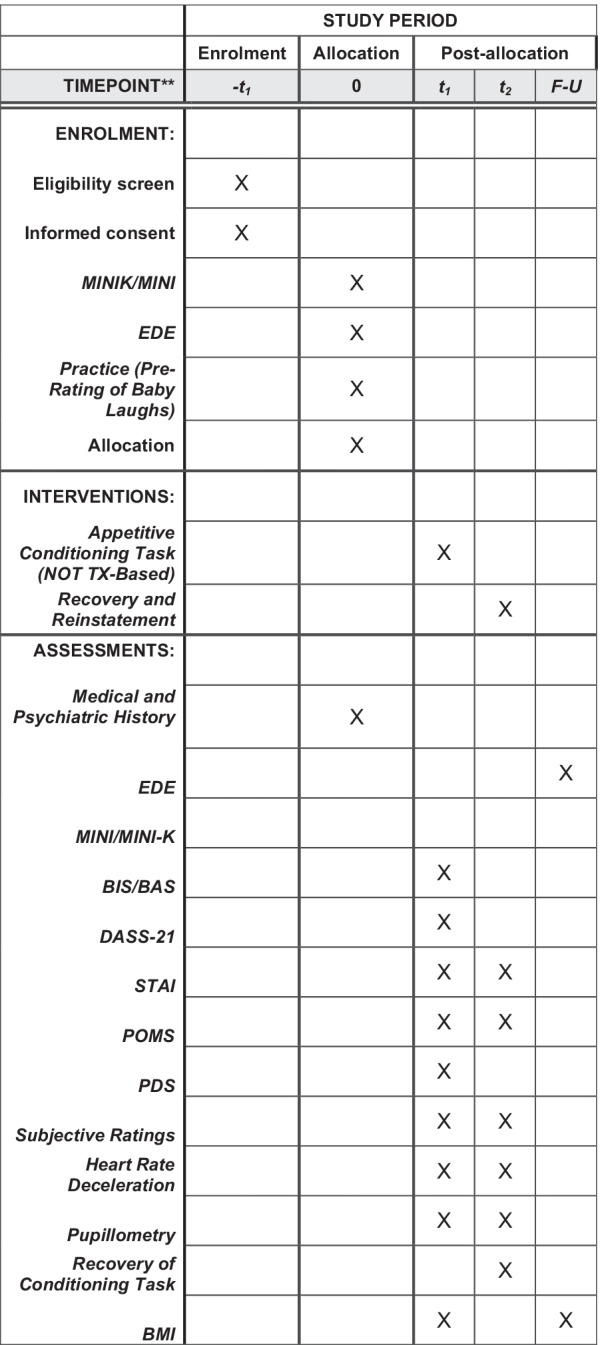
Schedule of enrollment, interventions, and assessments. *Recommended content can be displayed using various schematic formats. See SPIRIT 2013 Explanation and Elaboration for examples from protocols. **List specific timepoints in this row

The conditioning paradigm consists of the following phases: Day 1—(1) habituation, (2) acquisition, (3) extinction phase; Day 2—(4) spontaneous recovery, (5) reinstatement, (6) reinstatement test (See Fig. 1). Day 2 will occur 24 h after Day 1. CS presentation duration will be 5.73 s, with inter-trial intervals with fixation jittered between 1.9 and 11.5 s (mean 5.7 s) (see Fig. 2). The jittering across all trial phases was optimized for detection efficiency, with an exponential distribution of null events, using optseq2 (https://surfer.nmr.mgh.harvard.edu/optseq/). No more than three of the same CS will be presented sequentially. In habituation, two trials each of the CS+ and CS− will be presented, respectively. During acquisition, 20 presentations of the CS+ (75% paired with the US) and 20 presentations of the CS− (never paired with the US) will be presented (40 trials). During extinction, 20 exposures of each CS without the US will be presented (40 trials). Recovery will be assessed 24 h after the original appetitive conditioning procedure, and will consist of 20 exposures of each CS without the US (40 trials). Following this, the reinstatement paradigm will first consist of a 3-min exposure to a fixation cue, during which 4 uncued exposures to the US (infant laughter) will be delivered binaurally via noise canceling headphones (500 ms, 102 dBA). Subsequently, 20 exposures of each CS without the US will be presented (40 trials).

This appetitive conditioning paradigm did not incorporate ED-salient cues for several reasons. Importantly, appetitive conditioning paradigms are intended to assess the extent to which a previously benign cue can be associated with a positively experienced cue. The use of benign cues as the CS is especially important—such that no prior positive or negative association exists already, as this would contaminate the associative learning which the paradigm is intended to assess. If the cue used as the CS has existing associations, this may result in an assessment of recall or counter conditioning, rather than pure appetitive conditioning. ED-relevant cues may have likely already acquired valence or associations for those with AN, and so their use in an appetitive conditioning paradigm would corrupt the core theoretical tenets of conditioning paradigms. Moreover, this existing association to ED-relevant cues would likely not extend to control participants, which would therefore mean that the paradigm assesses different mechanisms in AN and control participants, respectively.

With regard to the US, this is required to be universally and comparably hedonic across both groups, so that the positive association can be transferred to the CS over repeated cue pairings. While the use of disorder-relevant stimuli as the US may have relevance for those with AN, it is unlikely that disorder- relevant cues deemed positive and hedonic by those with AN (i.e., thin images) would have comparable positive valence in those without AN. As such, this would not facilitate a meaningful between-group comparison of appetitive conditioning. As such, and in keeping with these important theoretical tenets, ED-salient cues will not be employed in the paradigm.

### Outcome measures

A summary and the timing of administration of outcome measures is outlined in Table 1.

**Table 1:** An overview of the timing and administration of outcome measures

Construct	Assessment	Visit		
		1	2	3
Medical stability	Medical history, vital signs for all participants. Physical exam/labs for low weight participants	X		
Reward processes	Behavioral Inhibition System/Behavioral Activation System Scale	X		
ED symptoms	Eating Disorder Examination Interview	X		
Neurodevelopment	Pubertal Development Scale	X		
Comorbidities	MINI/MINI-KID standardized diagnostic interview	X		
Depression	Depression Anxiety Stress Scale (DASS-21)	X		
Anxiety/mood	State-Trait Anxiety Inventory/Profile of Mood States (POMS-2 youth and adult short form)	X	X	
Appetitive learning	Appetitive Conditioning Task		X	
	Self-reported pleasure, valence, expectancy		X	
	Heart rate deceleration		X	
	Pupillometry		X	
	Task-based fMRI		X	
Recovery of appetitive conditioning	Recovery of Conditioning Task			X
	Self-reported pleasure, valence, expectancy			X
	Heart rate deceleration			X
	Pupillometry			X
	Task-based fMRI			X
Reinstatement of appetitive conditioning	Reinstatement of Conditioning Task			X
	Self-reported pleasure, valence, expectancy			X
	Heart rate deceleration			X
	Pupillometry			X
	Task-based fMRI			X

#### Primary outcome measures

*Subjective ratings* A 11-point Likert scale (− 5 to 5) will assess self-reported pleasantness and arousal for each CS, and US expectancy [74, 75]. Ratings will be measured at the end of every phase.

*Heart rate deceleration* Heart rate deceleration has been strongly associated with appetitive Pavlovian conditioning (η_p_^2^ of 0.8) [76]. Using a BioPac MP160 (Biopac, Goleta CA), we will measure heart rate deceleration/acceleration via heart period responses from each inter-trial interval to CS termination in both acquisition and extinction phases, and during the recovery and reinstatement phase. We will use a QRS detection algorithm and preprocess using PsychoPhysiological Modelling toolbox in Matlab (http://pspm.sourceforge.net/).

*Pupillometry* Increases in pupillary diameter have been associated with appetitive Pavlovian conditioning (η_p_^2^ of 0.29) [76], and with locus coeruleus activity [77, 78], which itself is associated with orienting of attention and reward anticipation [79]. Further, pupillary dilation is not correlated with HR deceleration [76], providing a proxy for central neurophysiology that is distinct from heart period responses. We will track each participants’ pupillary diameter and eye movements using a high precision MR-compatible EyeLink 1000 Plus (SR Research, Ottawa, Ontario Canada) eye tracker camera that can sample up to 2000 Hz. We will smooth the data using a median filter. Pupillary diameter data will be baseline corrected using the mean diameter in a time window of 2 s prior to CS onset. We will convolve the stimulus onsets with a canonical pupillary response function [80].

*fMRI acquisition and processing* We will use a GE Discovery 3T scanner with a 32-channel head coil. T1-weighted MPRAGE (0.8 mm^3^) using HCP Lifespan protocols (humanconnectome.org) will be used for registration and to measure gray matter volumes and thickness for exploratory covariates, as they can influence regional activation measures. Task functional data will be acquired using the HCP Lifespan multiband sequence: 2 mm^3^ and TR = 955 ms (https://www.cmrr.umn.edu/multiband), and spin echo field maps to correct for geometric distortions. Image Processing will be done with fMRIPrep [81]. Spatial normalization of the T1 image to standard MNI space will be performed through nonlinear registration. The BOLD timeseries with slice-timing correction will be resampled onto their native space by applying a single, composite transform to correct for head-motion and (estimated) susceptibility distortions. The BOLD timeseries will then be resampled into standard MNI space, generating the spatially-normalized, preprocessed BOLD runs. Automatic removal of motion artifacts using independent component analysis (ICA-AROMA) will be performed after spatial smoothing with a Gaussian kernel of 6 mm FWHM. We will use reward ROIs involved in appetitive conditioning [58] to test our hypotheses involving the nucleus accumbens (NAcc), and exploratory ROIs in the appetitive conditioning circuit [58] including the OFC, amygdala, ventral tegmental area (VTA), anterior cingulate cortex (ACC), and posterior cingulate cortex (PCC). Probabilistic ROIs will be obtained from the following atlases: OFC, ACC, PCC (Harvard–Oxford atlas); central and basolateral amygdala (Juelich Histological Atlas); NAcc, VTA (Reinforcement Learning Atlas [82]). Eigenvalues from the first eigenvariate of voxels within each ROI over the timeseries for the CS+ versus CS− contrast will be derived, indexing brain activation.

#### Baseline measures

*Medical history* All AN participants will be screened for medical stability, and any participant with acute medical instability will be excluded. In addition, participant height and weight will be measured for all participants, and all participants will be screened for general contraindications for MRI scanning procedures.

*Eating disorder examination* [71] The Eating Disorder Examination (EDE) is a widely-used clinician-administered diagnostic interview with excellent psychometric properties [71]. This interview assesses eating symptomatology over the preceding 28 days, and comprises four subscales; Dietary Restraint, Eating Concern, Shape Concern and Weight Concern. The EDE will be administered by licensed and EDE-trained mental health professionals with eating disorder expertise.

*MINI/MINI-KID diagnostic* *interview* [83] The Mini International Neuropsychiatric Interview (MINI 7.0.2) and Mini International Neuropsychiatric Interview for Children and Adolescents (MINI-KID) are robust and psychometrically strong structured diagnostic interviews for adults and children, respectively, for DSM and ICD psychiatric disorders [83]. The MINI and MINI-KID will be administered by licensed mental health professionals.

*Behavioral Inhibition Scale/Behavioral Approach Scale* [84] The Behavioral Inhibition Scale /Behavioral Approach Scale (BIS/BAS) is a measure of individual sensitivities in the drive to avoid potentially aversive outcomes and approach goal-oriented outcomes, showing good psychometric properties [84].

*Depression, Anxiety and Stress Scale* [85] The Depression, Anxiety and Stress Scale (DASS) is a widely used dimensional measure of anxiety, depression, and stress, which demonstrates strong psychometric properties and high internal consistency [86].

*State-Trait Anxiety Inventory* [87] The State-Trait Anxiety Inventory (STAI) is a self-report measure of state and trait anxiety, which has been widely used in both eating disorder [88] and conditioning research [89], and demonstrates excellent psychometric properties [87]. For a comprehensive overview of trial protocol and study measures, please see Additional file: 2 for a Spirit checklist.

### Data analysis

#### Power analysis

The sample is powered for the primary outcomes for our primary hypotheses based on an alpha of 0.0125 (corrected for 4 independent tests for each dependent variable) and accounting for the primary four phases of conditioning. For the whole-sample hypotheses, N = 90 total provides > 95% power to detect previously reported large effect sizes for heart rate and pupil dilation (η_p_^2^ = 0.8 and 0.29, respectively) [76], and NAcc CS+ versus CS− contrasts [69]. While effect sizes for appetitive conditioning (1) with infant laughter, and (2) in AN, are unknown, we have 86% power to detect a small effect size (η_p_^2^ = 0.042). If recruitment and enrollment goals are not met, for our primary hypothesis a total sample size of N = 72 (n = 24 in each of 3 groups) would be able to detect a medium effect size of η_p_^2^ = 0.06 with 89% power, while N = 60 (n = 20 in each of 3 groups) would be able to detect a medium effect size of η_p_^2^ = 0.06 with 80% power. For our hypotheses comparing AN-R and WRANR, a sample size of 60 provides > 95% power to detect similar large effect sizes for heart rate and pupillary dilation (η_p_^2^ = 0.29 to 0.8), 90% power to detect a medium effect size (η_p_^2^ = 0.06), and 72% power to detect a small effect size (η_p_^2^ = 0.042).

#### Analysis plan

Variable transformations prior to subsequent analyses will be determined based on distribution diagnostics and outlier analysis. Hypotheses (1), (3), and (4) will be tested using general linear models with between-subject factor of group (AN, WRAN; control), within-subject factors of stimulus (CS+ vs. CS−) and time (post-acquisition vs. post-extinction vs. spontaneous recovery vs. reinstatement), and their interactions. Covariates include age, age^2^, pubertal development scores, medication status, and depression symptom severity. These will be done separately for each of the dependent variables (Bonferroni-corrected): self-reported positive ratings, self-reported expectancy ratings, heart period response (specifically, operationalized primary deceleration [90]), pupillary response, and mean BOLD eigenvariate magnitude in the NAcc for the CS+ versus CS− contrast [69]. Exploratory analyses will be done with other appetitive conditioning ROIs (OFC, ACC, PCC, amygdala, and VTA), as well as for self-reported rating for pleasantness, arousal, and reward expectancy. To test hypothesis (2), we will use PROCESS v3.5 operated in SPSS, employing bootstrapping with 5000 resamples [91] to (separately) model moderation effects of (a) heart period response (primary deceleration) and (b) pupillary response on the relationship between NAcc eigenvariate magnitude for the CS+ versus CS− contrast (independent variable) and positive experience ratings (dependent variable).

## Discussion

The study proposed here will be the first systematic interrogation of appetitive conditioning in AN. Successful long-term treatment for AN hinges not only on a therapeutic ability to optimize approach behaviors to food consumption to halt the proximal, life-threatening symptoms related to starvation, but also to remediate the ability to respond hedonically to social and other stimuli that may be required to motivate one to recover from an otherwise chronic illness and stay in a remitted state. Without the help of the proverbial “carrot” of life enjoyment, the fear of the “stick” (weight gain, perceived fatness, and imperfection) will remain the dominant motivator of behaviors.

Existing studies have cast a varied picture of diminished or elevated reward responses in AN, depending on the stimulus, nutritional state, and likely other latent factors. Yet, critically, whether individuals can learn to respond to new rewarding stimuli has not been studied. This study will examine whether underweight or weight-restored individuals with AN can be conditioned to learn positive responses to non-symptom-related rewarding stimuli, as measured by subjective experience, physiology, and brain responses. Non-symptom-related cues—in this case, the socially rewarding sounds of infant laughter—allow us to examine the functional integrity of mechanisms underpinning appetitive learning. Further, these cues will mitigate the potentially confounding effects of learned associations that are linked to symptoms of AN.

Data obtained from the study will be used to develop a model (to be tested and validated in future data sets) to predict appetitive conditioning from physiological and neural variables. An additional next step would be to determine if symptom-related, potentially hedonic cues can be counter-conditioned—that is, if those previously aversively conditioned such as food can acquire positive valence. Results will help establish objective biomarkers of appetitive conditioning in AN and lay the groundwork for developing novel lines of treatment for AN and other psychiatric disorders involving diminished ability to experience pleasure and reward.

## Supplementary Information

**Additional file 1:** Pilot data.

**Additional file 2:** Spirit checklist.

## Data Availability

The datasets generated and analyzed during the current study contain clinical data and are not publicly available due to the protection of participants’ rights to privacy and data protection, but are available from SBM and JDF upon reasonable request. Summary data will be made available on ClinicalTrials.gov.

## Abbreviations

AN: Anorexia nervosa
CS: Conditioned stimulus
US: Unconditioned stimulus
