# Supplementary material for: Neural, physiological, and psychological markers of appetitive conditioning in anorexia nervosa: a study protocol
Source: J Eat Disord. 2022 May 10;10:68. doi: 10.1186/s40337-022-00546-5 (PMC9092702; doi:10.1186/s40337-022-00546-5)
Supplement: Supplementary file 1 — Additional file 1: Pilot data. [file 40337_2022_546_MOESM1_ESM.docx]

**Online Supplement**

**Pilot data**

In assessing hedonic cues for use in an appetitive conditioning paradigm in AN, and owing to mixed reports around the valence of sucrose in those with AN ^1^, we pilot tested alternative, symptom-neutral hedonic cues of smiling attractive faces and infant laughter vocalizations ^2^ in 11 adolescents and young adults with AN. Infant laughter resulted in mean positive ratings of 1.14±1.77 (scale -4 to +4), which were significantly higher than ratings for attractive faces of .26±1.07 (t=1.92, p=.042). In maximizing the valence of cues to be used during appetitive conditioning, and in accord with previous conditioning studies^3^, we extracted the four highest rated cues for each participant, which yielded a mean rating of 3±1.31. Importantly, these ratings were larger than ratings for cues used in previous appetitive conditioning studies (2.7±2.1)^3^, and for ratings of infant laughter cues in previous studies (1.21±1.51)^2^, where significant BOLD signal response in reward circuitry has been noted (Figure 2). This suggests that individuals with AN have an overall positive response to infant laughter, informing our cue selection for this study.

**Figure 1**: An overview of valence ratings for (i) infant laughter cues in previous studies, (ii) cues used in previous appetitive conditioning studies, (iii) attractive faces, (iv) infant laughter cues in the present study, and (v) the four highest rated infant laughter cues in the present study.


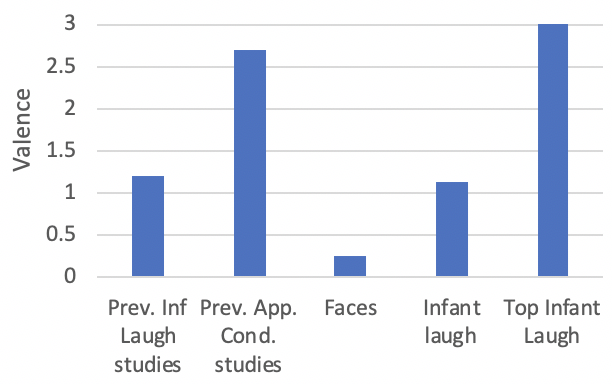


**References**

1. Murray SB, Strigo IA. Anorexia nervosa, neuroimaging research, and the contextual salience of food cues: The food approach-avoid conundrum. International Journal of Eating Disorders 2018; 51: 822-825.
2. Parsons CE, Young KS, Craske MG, Stein AL, Kringelbach ML. Introducing the Oxford Vocal (OxVoc) Sounds database: a validated set of non-acted affective sounds from human infants, adults, and domestic animals. Frontiers in Psychology 2014; 5: 562.
3. Stussi Y, Delplanque S,Coraj S, Pourtois G, Sander D. Measuring Pavlovian appetitive conditioning in humans with the postauricular reflex. Psychophysiology 2018; 8: e13073.
4. Riem MME, Van Ilzendoorn MH, Tops M, Boksem MAS, Rombouts SARB, Bakermans-Kraneburg MJB. No laughing matter: intranasal oxytocin administration changes functional brain connectivity during exposure to infant laughter. Neuropsychopharmacology 2012; 37: 1257-1266.
